# Relationships among autistic traits, depression, anxiety, and maternal–infant bonding in postpartum women

**DOI:** 10.1186/s12888-023-04970-y

**Published:** 2023-06-26

**Authors:** Naoki Fukui, Yuichiro Watanabe, Takaharu Motegi, Koyo Hashijiri, Maki Ogawa, Jun Egawa, Takayuki Enomoto, Toshiyuki Someya

**Affiliations:** 1 Department of Psychiatry, Niigata University Graduate School of Medical and Dental Sciences, 757 Asahimachidori-Ichibancho, Chuo-Ku, Niigata, 951-8510 Japan; 2 Department of Obstetrics and Gynecology, Niigata University Graduate School of Medical and Dental Sciences, Niigata, Japan

**Keywords:** Autistic traits, Maternal–infant bonding, Depression; anxiety, Parity; early postpartum

## Abstract

**Background:**

Although several studies have found significant relationships between autistic traits and depression/anxiety, the relationships between autistic traits and postpartum depression/anxiety remain unclear. Moreover, few studies have examined the relationships between autistic traits and mother–infant bonding while considering depression or anxiety.

**Methods:**

This study used a cross-sectional data analysis design. Participants were 2692 women who completed the Autism-Spectrum Quotient (AQ), Hospital Anxiety and Depression Scale (HADS), and Mother-to-Infant Bonding Scale (MIBS) at 1 month postpartum. We performed path analysis that included parity, the five AQ subscales (social skills, attention switching, attention to detail, communication, and imagination), both HADS subscales (anxiety and depression), and the two MIBS subscales (lack of affection and anger and rejection).

**Results:**

Our path analysis revealed that higher scores for social skills, attention switching, communication, and imagination were associated with higher scores for depression. Higher scores for social skills, attention switching, attention to detail, and communication were associated with higher scores for anxiety. Moreover, difficulties in social skills and imagination were associated with failure of maternal–infant bonding. However, more attention to detail was associated with better maternal–infant bonding.

**Conclusions:**

This study suggests that maternal autistic traits are related to anxiety and depression to a certain degree, but only slightly related to maternal–infant bonding at 1 month postpartum. To improve autistic women’s quality of life and that of their newborns, perinatal mental health issues such as anxiety, depression, and maternal–fetal bonding difficulties should be appropriately addressed.

## Introduction

Autism spectrum disorder is a neurodevelopmental disorder characterized by impaired social communication and interaction, as well as restricted interests and repetitive behaviors that significantly affect social functioning [1]. Although difficulties in social functioning in people with autism spectrum disorder have historically been considered solely individual deficits, recent work suggests that these difficulties may reflect bidirectional communication issues between autistic and non-autistic people [2]. Moreover, it has been recognized that autistic traits are continuously distributed across the general population [3]. Studies focused on the general population have reported significant relationships between autistic traits and mental health problems such as depression and anxiety [4, 5].

Perinatal depression and anxiety have been related to impaired maternal–infant bonding that may in turn lead to a lack of/delayed emotional response to the infant, feelings of irritability or hostility, aggressive impulses, or rejection of the child [6, 7]. In our recent study using the Mother-to-Infant Bonding Scale (MIBS) and the Hospital Anxiety and Depression Scale (HADS) for 2379 Japanese mothers (1116 primipara and 1263 multipara), depression and anxiety had negative influences on mother–infant bonding at 1 month after childbirth [8].

Furthermore, several recent studies have reported that mothers with a diagnosis of autism are more likely than non-autistic mothers to report experiencing prenatal/postnatal depression and anxiety [9–11]. There is also evidence that mothers with an autism diagnosis have higher rates of self-reported post-partum depression and anxiety symptomatology [12]. Moreover, a qualitative exploration survey reported that mothers with a diagnosis of autism were more likely than non-autistic mothers to face challenges such as a lack of social support, a lack of accessible parent and baby groups, and a lack of communication accommodation during interactions with healthcare providers [13]. Thus, it is possible that these factors are confounding factors between autistic traits and prenatal/postnatal depression and anxiety.

Although some studies have examined perinatal women with autism mentioned above, only two studies [14, 15] have examined the relationship between autistic traits and perinatal depression. The Japan Environment and Children’s Study [14] used the 10-item short version of the Autism-Spectrum Quotient (AQ) (AQ-10), the Edinburgh Postnatal Depression Scale (EPDS), and the short version of the MIBS. Logistic regression analyses revealed significant associations between the groups categorized by AQ-10 scores (0–1, 2, 3, 4–6, and 7–10 points) and postpartum depression (EPDS scores were ≥ 9 points) at 1 month postpartum. The authors also reported that mothers’ autistic traits were predictive of maternal–fetal bonding. The Hamamatsu Birth Cohort Study [15] used the Broader Phenotype Autism Symptoms Scale [16] and EPDS, and revealed the association between broader autism phenotype (BAP) and postpartum depression (EPDS scores were ≥ 9 points) during the first 3 months after childbirth.

In the current study, we used the full version of the AQ, which allowed us to access a larger amount of information for the AQ subscales, and the HADS, which has a sufficient number of anxiety items as well as depression items. Therefore, this study aimed to explore the relationships among autistic traits, depression, anxiety, and maternal–infant bonding in the early postpartum period.

In accord with a valuable suggestion by Sasson et al. [17], it should be noted that the current study did not examine individuals with autism, but autistic traits in a general population of postpartum women.

## Methods

### Ethics statement

We have conducted the Perinatal Mental Health Research Project involves the Niigata University Medical and Dental Hospital, Department of Obstetrics and Gynecology and 33 associated obstetric institutions in Niigata Prefecture. This study was part of the project approved by the Niigata University Ethics Committee (approval number: 2016–0019). Written informed consent was obtained from all participants. Study procedures were carried out in accordance with the principles of the Declaration of Helsinki.

### Participants

We started the project in March 2017 [8, 18–21] and collected data using the Japanese versions of the Autism-Spectrum Quotient (AQ), HADS, and MIBS for 2692 postpartum women (1263 primipara and 1429 multipara, mean age 31.6 ± 4.8 years) who visited the participating obstetric institutions at 1 month postpartum. Participants included the same cohort as used in our previous study [8, 18–21]. The current study used a cross-sectional data analysis design. We excluded women with serious physical complications and serious pregnancy complications from this study. Furthermore, women who currently had regular clinic or hospital visits to receive psychotherapy and/or medication for psychiatric disorders (e.g., autism spectrum disorder, schizophrenia, depression, bipolar disorder, anxiety disorder, or personality disorder) were excluded from this study. Although women with undiagnosed autism spectrum disorder could be included under this exclusion criterion, the prevalence of autism spectrum disorder is considered to be approximately 1% and the male-to-female ratio is 3:1 [22]. Thus, the current study was focused on autistic traits rather than autism spectrum disorder, and this allowed us to obtain a larger sample.

### Measurements

The AQ is a standardized, self-report questionnaire that measures the extent to which adults of normal intelligence possess characteristics associated with the autism spectrum [23]. The AQ has 50 items on five subscales: social skills, attention switching, attention to detail, communication, and imagination. The AQ uses a forced-choice response format, with respondents asked to rate each statement as “definitely agree,” “slightly agree,” “slightly disagree,” or “definitely disagree.” Ratings of agree (definitely or slightly) are scored as “1” and ratings of disagree (definitely or slightly) are scored as “0.” Higher scores indicate higher autistic traits. AQ scores ranged from 0 to 50 points. Ruzich et al. performed a systematic review and reported that AQ was useful for evaluating autistic traits, not only in typical adult males and but also typical adult females [24]. The AQ was translated into Japanese by Wakabayashi et al., who showed internal consistency using Cronbach’s alpha values as follows: social skills = 0.78, attention switching = 0.63, attention to detail = 0.57, communication = 0.64, imagination = 0.51 [25]. We also calculated the Cronbach’s alpha values for AQ subscales, as follows: social skills = 0.73, attention switching = 0.57, attention to detail = 0.61, communication = 0.69, and imagination = 0.46. A recent Japanese study examining the relationship between autistic traits and perinatal depression used the AQ-10 mentioned above [14]. As the AQ-10 only includes a few items assessing each subscale, the reliability of each subscale cannot be guaranteed. Therefore, we used the full version of the AQ to obtain more informative data on maternal autistic traits.

In clinical settings, the HADS has been commonly used in order to screen for anxiety and depression [26]. The scale comprises 14 items: seven items that assess anxiety (items 1, 3, 5, 7, 9, 11, and 13) and seven items that assess depression (items 2, 4, 6, 8, 10, 12, and 14). Participants are asked to rate the 14 items on a four-point Likert-type scale from 0 to 3. HADS scores ranged from 0 to 42 points. The HADS was previously translated into Japanese by Kitamura [27], and the Japanese version was validated in a previous study [28]. In this study, Cronbach’s alpha values for anxiety and depression were 0.81 and 0.85, respectively.

Kumar and colleagues [29] developed the Mother–Infant Bonding Questionnaire (MIBQ) to evaluate bonding disturbance during the postpartum period. They subsequently revised the MIBQ to develop the MIBS [30]. The MIBS underwent further modification changing the wording of a few items and adding a new item by Marks and colleagues. Yoshida and colleagues translated this version of the MIBS into Japanese [31] as the MIBS-J. We previously described the MIBS-J in detail [18]. Participants are asked to rate the 10 MIBS-J items on a four-point Likert-type scale from 0 to 3. MIBS scores ranged from 0 to 30 points. A two-factor structure: lack of affection (items 1, 6, 8, and 10) and anger and rejection (items 2, 3, 5, and 7) was found in our previous study [18]. Therefore, we used these two factors as subscales in this study. In this study, Cronbach’s alpha values for lack of affection, and anger and rejection, were 0.73 and 0.71 respectively.

Obstetric characteristics such as parity (primiparous or multiparous), type of conception (natural conception or others), gestational age at delivery (full term or preterm delivery) and type of delivery (vaginal delivery or cesarean section) were obtained through interviews by midwives.

### Missing data

Among the data of 3,098 participants, we excluded that of 406 participants with missing data for AQ (*n* = 305), HADS (*n* = 79), MIBS (*n* = 20), parity (*n* = 34), type of conception (*n* = 20), gestational age at delivery (*n* = 28) and type of delivery (*n* = 18).

### Statistical analysis

We used dummy variables as follows: parity (primiparous = 1, multiparous = 2), type of conception (natural conception = 1, others = 2), gestational age at delivery (full term = 1, preterm delivery = 2) and type of delivery (vaginal delivery = 1, cesarean section = 2). We defined preterm as the birth at fewer than 37 weeks.

First, on the basis of a previous study [32], we divided our sample into three groups with different levels of AQ scores, as follows: normal, broader autism phenotype (BAP; AQ scores of 1 to 2 SDs above the mean) and medium autism phenotype (MAP; AQ scores of 2 SDs above the mean). Comparisons were made among these three groups for sample characteristics, HADS scores, and MIBS scores. We used chi-square tests for categorical data and analysis of variance for continuous variables. The level of significance was set at *p* < 0.005 according to the Bonferroni correction of eight statistical tests.

Second, we calculated the correlation coefficients for obstetric characteristics and the AQ, HADS, and MIBS-J subscale scores. The level of significance was set at *p* < 0.00064 based on Bonferroni correction of 78 statistical tests.

Third, we performed a path analysis that included parity, the five AQ subscales (social skills, attention switching, attention to detail, communication, and imagination), both HADS subscales (anxiety and depression), and the two MIBS-J subscales (lack of affection and anger and rejection). Because a previous study reported that autistic traits tend to be stable over time [33], we assumed that autistic traits would precede perinatal anxiety, depression, and mother-infant bonding. Regarding the causal relationship between anxiety/depression and maternal-infant bonding, another study examined subjects diagnosed with postpartum onset of mental disorders, such as psychosis, mania, and depression [34]. The results revealed that the majority of subjects experienced improvement of maternal-infant bonding in accord with improvement of mental disorders by pharmaceutical treatment [34]. Because there is considered to be a continuum between diagnosed depression and depressive symptoms in the general population, we assumed that perinatal anxiety/depression would precede mother-infant bonding. In concrete terms, these analyses were as follows. [1] We drew paths from each of the five AQ subscales to the two HADS subscales. [2] We then drew paths from each of the five AQ subscales to the two MIBS-J subscales. [3] Next, we drew paths from the two HADS subscales to the two MIBS-J subscales. [4] Finally, we drew paths from parity to the two HADS subscales and the two MIBS-J subscales. After these analyses, we retained statistically significant paths (*p* < 0.05). We used the comparative fit index (CFI) and the root mean square error of approximation (RMSEA) to evaluate the goodness of fit (CFI ≥ 0.95 and RMSEA ≤ 0.06) [35] between the models and the data. All statistical analyses were conducted using SPSS version 25 (IBM Corp., Armonk, NY, USA) and Amos 25.0.0 (IBM Japan, Tokyo, Japan).

## Results

### Comparisons among three phenotypes defined by autism-spectrum quotient scores

For the increasing range of AQ scores, in the normal, BAP, and MAP groups, the mean scores for HADS total, HADS anxiety, HADS depression, MIBS total, MIBS lack of affection and MIBS anger and rejection also increased significantly (Table 1).

**Table 1:** Sample characteristics, Hospital Anxiety and Depression Scale, and Mother-to-Infant Bonding Scale scores

	Phenotypes defined by AQ			Statistics
	Normal	BAP*	MAP**	
AQ score range	0 – 23	24 – 30	31 –	
Number	2239	369	84	
Sample characteristics				
Primiparous / multiparous (n)	1033 / 1206	189 / 180	41 / 43	χ^2^ = 3.41, *P* = 0.18
Natural conception / others (n)	1984 / 224	329 / 37	78 / 6	χ^2^ = 0.81, *P* = 0.67
Full term / preterm delivery (n)	2151 / 60	353 / 12	77 / 6	χ^2^ = 5.91, *P* = 0.052
Vaginal delivery / caesarean section (n)	1864 / 363	306 / 59	72 / 12	χ^2^ = 0.24, *P* = 0.89
HADS				
Total mean scores [SD]	10.95 (6.00)	14.98 (6.71)	17.35 (6.73)	F = 105.56, *P* < 0.001^#^
Anxiety mean scores [SD]	5.06 (3.60)	7.26 (4.15)	8.40 (4.13)	F = 83.17, *P* < 0.001^#^
Depression mean scores [SD]	5.89 (3.24)	7.73 (3.57)	8.94 (3.62)	F = 78.28, *P* < 0.001^#^
MIBS				
Total mean scores [SD]	1.98 (2.41)	3.24 (3.19)	3.76 (3.06)	F = 54.60, *P* < 0.001^#^
Lack of affection mean scores [SD]	0.93 (1.43)	1.50 (1.79)	1.86 (1.94)	F = 35.69, *P* < 0.001^#^
Anger and rejection mean scores [SD]	0.33 (0.83)	0.66 (1.27)	0.79 (1.19)	F = 28.37, *P* < 0.001^#^

*Abbreviations*: *AQ* Autism-Spectrum Quotient, *BAP* Broader Autism Phenotype, *MAP* Medium Autism Phenotype, *HADS* Hospital Anxiety and Depression Scale, *MIBS* Mother-to-Infant Bonding Scale, SD standard deviation

BAP* is defined as AQ scores of 1 to 2 SDs above the mean. MAP** is defined as AQ scores of 2 SDs above the mean [30]

^#^Level of significance set at *P* < 0.005 based on Bonferroni correction of 10 statistical tests

### Correlation analysis

Table 2 shows the correlation coefficients between each item. As shown in our previous study [21], only parity (among the obstetric factors) was associated with psychological factors (i.e., attention switching, anxiety, lack of affection, and anger and rejection scores) (Table 2). Therefore, we included parity in the subsequent path analyses.

**Table 2:** Correlation coefficients for sample characteristics and Autism-Spectrum Quotient, Hospital Anxiety and Depression Scale, and Mother-to-Infant Bonding Scale scores

	1	2	3	4	5	6	7	S	9	10	11	12	13
(Sample characteristics)													
1. Primiparous / multiparous													
2. Natural conception / others	-.133**												
3. Full term / preterm delivery	-.011	.049											
4. Vaginal delivery / caesarean section	.011	.122**	.020										
(AQ)													
5. Social skills	-.029	-.001	.032	-.014									
6. Attention switching	-.073**	-.016	.018	-.026	.513**								
7. Attention to detail	-.061	-.019	-.019	-.002	-.128**	.033							
8. Communication	-.061	-.035	.032	-.017	.600**	.573**	-.039						
9. Imagination	-.052	.004	-.007	.007	.427**	.350**	-.074**	.458**					
(HADS)													
10. Anxiety	-.231**	.051	.024	.001	.220**	.286**	.101**	.266**	.162**				
11. Depression	-.057	.031	.001	-.004	.267**	.232**	-.028	.272**	.204**	.423**			
(MIBS)													
12. Lack of affection	-.132**	.030	.031	.027	.212**	.155**	-.050	.166**	.169**	.373**	.423**		
13. Anger and rejection	-.117**	.003	-.013	-.034	.158**	.121**	-.015	.124**	.100**	.282**	.236**	.236**	.401**

*Abbreviations*: *AQ* Autism-Spectrum Quotient, *HADS* Hospital Anxiety and Depression Scale, *MIBS* Mother-to-Infant Bonding Scale, *SD* standard deviation

^**^Level of significance set at *P* < 0.00064 based on Bonferroni correction of 78 statistical tests

### Path analyses

**Fig. 1:**
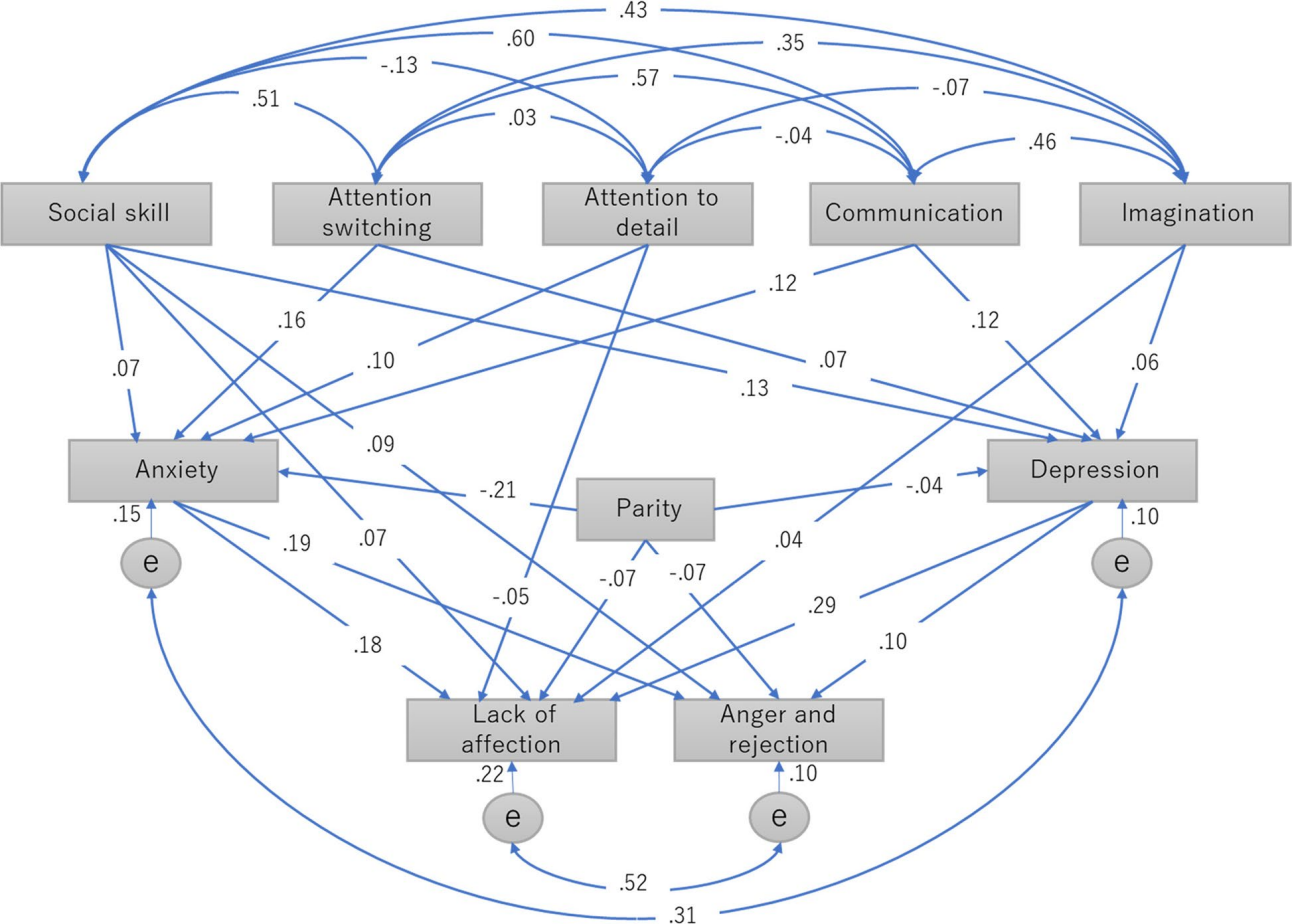
Path model of the relationships between the AQ, HADS, and MIBS subscales and parity. Abbreviations: AQ, Autism-Spectrum Quotient; HADS, Hospital Anxiety and Depression Scale; MIBS, Mother-to-Infant Bonding Scale

Figure 1 shows the path model that described the interactions among parity, the five AQ subscales, the two HADS subscales, and the two MIBS subscales. The paths shown in Fig. 1 indicate statistically significant relationships. The model had a good fit to the data (CFI = 0.996, RMSEA = 0.026). If the number presented with a path is positive, the higher the score of the factor where the path starts, and the higher the score of the factor to which the path is directed. We focused on the negative values listed in the paths, as explained below. Primiparous women tended to have higher scores for anxiety, depression, lack of affection and anger and rejection compared with multiparous women. Higher scores for attention to detail were related to lower scores for lack of affection.

## Discussion

The current study revealed an association between autistic traits and perinatal mental health in the general population, using a large sample size and multivariate analysis that allowed us to detect associations among multiple factors. These findings will provide useful information for informing the further development of perinatal mental health research and improvement of health care services for perinatal women.

In our path analyses, autistic traits as evaluated by the AQ were significantly associated with depression and anxiety at 1 month postpartum. Two previous Japanese studies [14, 15] mentioned above and our study showed similar results regarding the relationships between autistic traits and depression among postpartum women. Moreover, using the HADS in our study enabled us to detect a significant association between autistic traits and anxiety in postpartum women. To our knowledge, no previous study has investigated the relationship between autistic traits and anxiety in the postpartum period.

The Japan Environment and Children’s Study [14] used four AQ-10 subscales: social skills (three items); communication (two items); imagination (four items); and attention switching (one item). They reported significant associations between these AQ-10 subscales and postpartum depression. However, as we used the full-version of the AQ, we accessed a larger amount of information for the AQ subscales and detected more detailed associations between each AQ subscale and postpartum depression/anxiety. In concrete terms, we found that at 1-month postpartum, higher scores for social skills, attention switching, communication, and imagination predicted higher scores for depression, and higher scores for social skills, attention switching, attention to detail, and communication predicted higher scores for anxiety. For perinatal women with higher scores for social skills, a proactive approach by maternity staff and community health workers that provides guidance on management of pregnancy, delivery, and childcare may be effective to reduce depression and anxiety. For women with higher scores for attention switching or attention to detail, identifying important checkpoints in the management of pregnancy, delivery, and childcare may be effective to reduce depression or anxiety. For those with higher scores for communication, a proactive approach by maternity staff and community health workers focused on helping them avoid isolation may be effective in reducing depression and anxiety. Finally, for women with higher scores for the imagination subscale, helping them to visualize their management of pregnancy, delivery, and childcare may be effective to reduce depression.

This study offered interesting findings in that social skills, imagination, and attention to detail were differentially associated with maternal–infant bonding. Davis et al. investigated the relationships among the AQ subscales, looking at eyes, and face identity recognition ability in a general population [36]. They reported that more attention to detail was indirectly related to better face recognition via an increased number of fixations on the eyes during face learning. Conversely, there was an inverse trend whereby the higher the AQ social characteristics (social skills, attention switching, communication, and imagination), the lower the face recognition ability [36]. A mother’s emotional bonding with her infant is considered important to ensure the infant’s survival and healthy psychosocial development [37]. Human infants are born with facial features and other physical and behavioral characteristics that make adults want to approach and care for them [38]. Consequently, perinatal women with poor social skills and more attention to detail may tend to have a higher face recognition ability via an increased number of fixations of the eyes on their infant. This higher face recognition ability may help to establish better maternal–infant bonding. Moreover, a previous study of mothers with autism reported that some autistic mothers felt that their sensory abilities might allow them to better attend to and interpret their infant’s cues [38]. Additionally, there is some evidence that their child may represent a special interest for some autistic mothers [13]. This increased attention to their child could be beneficial for bonding.

Although attention to detail was found to improve maternal–infant bonding as mentioned above, we found this factor was also associated with higher anxiety at 1 month postpartum. It is difficult to determine why attention to detail had significant effects on anxiety, because few studies involving the general population used the full version of the AQ and examined relationships among the AQ subscales and anxiety. Barnett et al. [39] used the full-version of the AQ and the HADS to examine disordered eating (*N* = 691). They calculated the correlation coefficients for the AQ and HADS subscale scores and found significant associations between the five AQ subscales and anxiety/depression. They reported the correlation coefficient between attention to detail and anxiety as 0.197 (*p* < 0.001). Other studies that used a short form version of the AQ (excluding the attention to detail subscale) showed significant associations between other AQ subscales and depression in the general population, such as among workers [40] and perinatal women [14]. Further studies using the full version of the AQ are needed to confirm the relationships among autistic traits evaluated by five AQ subscales and depression/anxiety in the general population, especially among perinatal women.

As discussed above, the current results only demonstrated a relationship between autistic traits and anxiety/depression or maternal–fetal bonding. It has been reported that autistic parents face additional challenges, such as a lack of social support, a lack of accessible parent and baby groups, a lack of communication accommodation during interactions with healthcare providers [13], and sensory processing difficulties [41]. One of the factors that is considered to cause these problems in communication and sensory processing is a lack of knowledge about autism spectrum disorder among healthcare providers involved in the perinatal period. Therefore, some autistic mothers have advocated the importance of clear and direct communication and appropriate care regarding sensory experiences (e.g., auditory, tactile, body awareness, smell, vision) related with pregnancy, labor, birth, and breastfeeding by healthcare providers with sufficient knowledge about autism spectrum disorder [13, 41]. It has also been reported that income, which is potentially involved in a range of social, psychological, and health outcomes, is negatively associated with autistic traits in the general population [42]. Consequently, further studies dealing with the various factors mentioned above will be needed to identify confounding factors between autistic traits and perinatal mental health. If there are confounding factors, it would be impossible to identify the true relationship between autistic traits and perinatal mental health without adjusting for the influence of those factors. Besides research, in clinics, appropriate care for these potential confounding factors can contribute to the prevention of anxiety, depression, bonding difficulties and psychological trauma in perinatal women with high autistic traits or diagnosed autism spectrum disorder.

Our study had several limitations that merit discussion. First, this study used a cross-sectional design at 1 month postpartum. Further studies will be needed to examine how autistic traits are related to longitudinal changes in anxiety/depression and mother–infant bonding from pregnancy to several months or several years postpartum. Second, as we used self-report questionnaires, we could not determine the actual prevalence of autism spectrum, depressive, anxiety, and bonding disorders at 1 month postpartum. Third, we did not have data on additional factors such as social support, history of childhood maltreatment, education level, income, well-informed pregnancy conditions, marital/relationship status, and the infants’ sex, all of which are considered to be associated with anxiety/depression or maternal–infant bonding. Because factors other than infants’ sex may also have causal relationships with autistic traits, further studies will be needed to account for the influence of these factors. Fourth, although we recruited participants from 34 obstetric institutions in Niigata Prefecture, they may not be representative of the general population of postpartum women in Japan. Fifth, we used the original five subscales of AQ developed by Baron-Cohen et al., the Cronbach’s alpha values of which were relatively low, in accord with our results. Thus, further studies using AQ subscales identified by reliable factor analyses may be needed to explore the associations between autistic traits and perinatal mental health in more detail. Sixth, the scores for MIBS subscales were very low, indicating that most participants did not have maternal–infant bonding difficulties. Thus, it may be difficult to apply the results of this study to clinically meaningful maternal–infant bonding disability. Seventh, all of the coefficients related to AQ subscales in our path analysis had small values. Therefore, the association between autistic traits and perinatal mental health should not be overestimated on the basis of the current findings. Eighth, because this study did not target autism spectrum disorder, but autistic traits in a general population of postpartum women, the results of this study may not apply to postpartum women with autism spectrum disorder. Finally, it has been reported that the AQ has low to fair sensitivity and specificity as a diagnostic measure of autism spectrum disorder [43, 44]. Therefore, we should not overestimate the ability of the AQ to capture the lived experience of autistic people.

## Conclusions

The present study suggests that maternal autistic traits are related to anxiety and depression to a certain degree, but only weakly related to maternal–infant bonding at 1 month postpartum. Interestingly, attention to detail, in contrast to social characteristics such as social skill and imagination, was associated with better maternal–infant bonding.

To improve the quality of life of autistic women and their newborns, perinatal mental health issues such as anxiety, depression and maternal–fetal bonding difficulties should be appropriately addressed. As a preliminary step in this process, it is important to create an environment that facilitates access to evaluation of the qualities and quantity of autistic traits, whether the diagnostic criteria for autism spectrum disorder are met, and the symptoms of autism. Additionally, it is important that these efforts are undertaken to prevent the worsening of symptoms of autism that are likely to worsen when faced with stressful situations and emotional disturbances.

## Data Availability

All data generated or analysed during this study are included in this published article.

## Abbreviations

AQ: Autism-Spectrum Quotient
HADS: Hospital Anxiety and Depression Scale
MIBS: Mother-to-Infant Bonding Scale (Japanese version: MIBS-J)
AQ-10: 10-Item short version of the Autism-Spectrum Quotient
MIBQ: Mother–Infant Bonding Questionnaire
CFI: Comparative fit index
RMSEA: Root mean square error of approximation
BAP: Broader autism phenotype
EPDS: Edinburgh Postnatal Depression Scale
MAP: Medium autism phenotype
